# Facilitation and Competition among Invasive Plants: A Field Experiment with Alligatorweed and Water Hyacinth

**DOI:** 10.1371/journal.pone.0048444

**Published:** 2012-10-30

**Authors:** Emily J. Wundrow, Juli Carrillo, Christopher A. Gabler, Katherine C. Horn, Evan Siemann

**Affiliations:** Department of Ecology and Evolutionary Biology, Rice University, Houston, Texas, United States of America; United States Department of Agriculture, United States of America

## Abstract

Ecosystems that are heavily invaded by an exotic species often contain abundant populations of other invasive species. This may reflect shared responses to a common factor, but may also reflect positive interactions among these exotic species. Armand Bayou (Pasadena, TX) is one such ecosystem where multiple species of invasive aquatic plants are common. We used this system to investigate whether presence of one exotic species made subsequent invasions by other exotic species more likely, less likely, or if it had no effect. We performed an experiment in which we selectively removed exotic rooted and/or floating aquatic plant species and tracked subsequent colonization and growth of native and invasive species. This allowed us to quantify how presence or absence of one plant functional group influenced the likelihood of successful invasion by members of the other functional group. We found that presence of alligatorweed (rooted plant) decreased establishment of new water hyacinth (free-floating plant) patches but increased growth of hyacinth in established patches, with an overall net positive effect on success of water hyacinth. Water hyacinth presence had no effect on establishment of alligatorweed but decreased growth of existing alligatorweed patches, with an overall net negative effect on success of alligatorweed. Moreover, observational data showed positive correlations between hyacinth and alligatorweed with hyacinth, on average, more abundant. The negative effect of hyacinth on alligatorweed growth implies competition, not strong mutual facilitation (invasional meltdown), is occurring in this system. Removal of hyacinth may increase alligatorweed invasion through release from competition. However, removal of alligatorweed may have more complex effects on hyacinth patch dynamics because there were strong opposing effects on establishment versus growth. The mix of positive and negative interactions between floating and rooted aquatic plants may influence local population dynamics of each group and thus overall invasion pressure in this watershed.

## Introduction

Positive interactions among species are recognized as central drivers in structuring communities [1]–[4] and facilitation is increasingly recognized as a determinant of the invasive success of exotic species [5]–[7]. For example, exotic species may become invasive due to facilitative or mutualistic interactions with native species [8]–[10], may themselves facilitate native species [11], or may facilitate other exotics, which can increase their rates of establishment and growth [12]–[14]. In contrast, negative interactions between species can shape community structure as well [15]–[17]. For instance, the presence of certain native or exotic species can decrease the likelihood of future invasions of prey or competitors of those species [18], [19]. This is comparable to biotic resistance of invasions by native species [20], [21], and together with facilitative interactions, these forces can determine the composition of species in a community [22].

It has been observed that many ecosystems that are heavily invaded by an exotic species often contain several common exotic species [20], [23], and particular combinations of exotic species may co-occur frequently, which suggests that their distributions are not independent [20], [23], [24]. This could reflect independent responses to a single set of conditions, such as salinity or nutrient levels [25], or common pathways of introduction (such as from ballast water [26]), or it could be driven by facilitation or mutualistic interactions between exotic species, where the invasion of one species may make the subsequent invasion of one or more other exotic species more likely. This process of facilitation has been termed “invasional meltdown” in extreme cases because it has the potential to lead to an exotic species dominated ecosystem if such positive feedbacks between the initial invader and subsequent introduced species are sufficiently common or strong [20], [27].

Three types of facilitation among non-native species have now been described: simple facilitation, mutual facilitation, and invasional meltdown [20], [27]. The two weaker types of facilitation are: simple facilitation, in which one species aids the invasion of other species, resulting in an overall increase in the net invasion; and mutual facilitation, in which multiple invasive species reciprocally aid each other. These types of facilitation are population processes and are not referred to as a meltdown until they become community level processes. An invasional meltdown is defined by positive interactions between invasive species in which the net effect of interactions leads to an accelerating replacement of native communities by an increasing rate of establishment of invasive species after an initial population of an exotic species is established [27]. One classic example of invasional meltdown is that of the yellow crazy ant (*Anoplolepis gracilipes*) whose introduction to Christmas Island led to an increase in the abundance of previously introduced scale insects, which had until that point maintained relatively small populations, leading to canopy dieback and even death of native tree species [28].

It is also possible that some exotic species limit the establishment or abundance of other exotic species [19]. For instance, if one exotic species preys on another, the presence of the predator may limit the populations of the exotic prey species [19]. In addition, competition between two exotic species can occur with one species limiting the material, substance, or space of the other by occupying the same habitat or utilizing a scarce resource [29], [30]. This is analogous to biotic resistance to invasions due to interactions with native species [20], [21]. In these scenarios, it is possible that the overall extent of invasion is insensitive to these interactions even though the interactions may have strong effects on exotic species composition.

In order to further our understanding of invasions, it is necessary to understand the variety of positive and negative interactions among the species in an invaded environment. We currently have a wealth of information on exotic species and their effects on the environment [21]. However, in order to effectively manage exotic species where numerous exotics persist, it is imperative that we have an understanding of the interactions among exotic species and the effects of each on the invasion success of others [12], [31]. For example, removal of an exotic plant species that competes with another exotic species may simply result in a change in the dominant exotic plant species with no increase in the abundance of native species. In contrast, removal of an exotic species that facilitates the invasion of other species may be an extremely effective method of control that is not appreciated or undertaken because the relationships among exotic species are not fully understood [23], [32].

### Focal Species

Native to South America, alligatorweed [*Alternanthera philoxeroides* (Mart.) Griseb.], water hyacinth [*Eichhornia crassipes* (Mart.) Solms-Laubach], and water lettuce (*Pistia stratiotes* L.) each had broadly invaded freshwater habitats in North America by the late 1800’s [33]–[35]. Alligatorweed tends to form mats than can survive on land and in the water [33], [36]. These mats are rooted at the shoreline and can grow meters into the open water. Water lettuce and water hyacinth are both free-floating plants that drift by wind and current [34], [35]. All of these invasive plants are clonal reproducers, which aids in their spread and invasion. These three plants are model organisms for the study of invasional meltdown because they thrive in their non-native environment, can reproduce rapidly, and often grow in the same ecosystems thus increasing the probability of finding them together. Moreover, all three co-occur in the native range as well [37].

In the Armand Bayou watershed (see below for full site description), alligatorweed, water hyacinth, and water lettuce are each abundant and they are frequently found to occur together in a local area. Alligatorweed may facilitate water hyacinth and water lettuce invasion (simple facilitation) when the floating plants are driven by wind or currents into alligatorweed mats where they become entangled. In this mechanism, alligatorweed acts as an anchor for the floating plants and creates a nucleus of invasion. This could lead to rapid reproduction of the clonal floating plant species in areas where alligatorweed is established. In the absence of such trapping by anchored plants, these floating plants may reach Galveston Bay where they die from exposure to seawater and are lost from the population. Further, this could also reflect facilitation of alligatorweed invasion by water hyacinth and water lettuce (mutual facilitation) if alligatorweed mats then expand. As the initial colonists of these floating clonal species duplicate rapidly, alligatorweed may grow over and under the water hyacinth and/or water lettuce, trapping the two floating species in its roots, and thus creating a locally more extensive and persistent alligatorweed invasion.

To test whether there are positive or negative interactions among these exotic plant species in Armand Bayou, we performed an experiment in which we removed rooted (alligatorweed) and/or free-floating aquatic plant species (primarily water hyacinth but also water lettuce). We analyzed the establishment of new patches and expansion of existing patches separately to anticipate the possibility that interactions may have different effects on each process. This allowed us to quantify how the presence or absence of one plant functional group altered the likelihood of invasion or the intensity of invasion by species of the other functional group. That is, we could determine whether particular exotic species make subsequent invasions by other exotics more or less likely.

## Methods

### Study Site

We conducted this experiment in the upper tidal region of Armand Bayou in Pasadena, TX, which empties into Clear Lake, which in turn drains to Galveston Bay. Until the 1950’s, Armand Bayou (then Middle Bayou) was a meandering bayou with wetlands along its banks. Removal of groundwater, oil, and gas caused meters of subsidence in this area in the 1960’s and 1970’s. This caused the lower reaches to widen, eliminating the meanders and the bordering wetlands. Today, the water level fluctuates more than a meter within a month due to tides, rainfall, and prevailing winds that drive water into or out of Clear Lake. All necessary permits were obtained for the described field studies (TX Parks and Wildlife).

Armand Bayou is heavily invaded by several exotic aquatic plant species including alligatorweed, water hyacinth, and water lettuce. Alligatorweed occurs along the banks in addition to growing out into open water. Other invasive terrestrial plant species including Chinese privet (*Ligustrum sinense* Lour.), Chinese tallow tree [*Triadica sebifera* (L.) Small], hairypod cowpea [*Vigna luteola* (Jacq.) Benth.], and giant reed (*Arundo donax* L.) occur along the shores but were not the focus of this study.

### Initial Setup

In September 2007, we established sixteen plots that consisted of three meters of shoreline and the aquatic vegetation in the area defined by two parallel lines perpendicular to the average shoreline angle at the plot edges out into open water. Criteria for selecting plot locations were as follows: 1) both alligatorweed and water hyacinth or water lettuce had to be present, 2) the aquatic vegetation had to extend at least one meter but no more than three meters from the shoreline, 3) water depth at the outer edge of the aquatic vegetation mat could not exceed 1.5 meters, and 4) all plots had to be at least 50 meters apart. Because water depth fluctuates widely and was high when plots were established, we defined the shoreline as the place where non-emergent terrestrial plant species first occurred even if they were underwater at that time. We delineated plots with four PVC stakes spaced one meter apart in a line that approximated the average shoreline angle. The inner pair of stakes defined the data collection area.

We assigned each of the sixteen plots to two treatments in a completely randomized, factorial design. The first treatment was for presence of exotic rooted aquatic plants (alligatorweed removal or control), and the second treatment was for presence of exotic floating aquatic plants (water hyacinth and water lettuce removal or control). We removed plants by hand from the entire three-meter plot width for removal plots, carefully leaving behind all native plant species and any exotic species that were not targeted for removal in that plot. After removing the target invasive plant species, we bagged them and brought them to shore for proper disposal. This removal procedure occurred six times in order to maintain the treatments throughout the course of the experiment over eight weeks.

### Data Collection

We collected data on aquatic vegetation extent (including the area of open water within the vegetated area) and plant community composition every seven to ten days on the same day as maintenance of removal treatments. Our design allowed us to collect data on the growth of hyacinth and water lettuce and the establishment of alligatorweed in alligatorweed removal plots, as well as growth of alligatorweed and establishment of water hyacinth and water lettuce in hyacinth and lettuce removal plots. Data were always collected prior to the removal of plants for treatment maintenance.

We used a PVC measuring frame to define the one meter wide data collection area. These areas extended from the interior pair of PVC stakes (to buffer against edge effects), perpendicularly from the shoreline to the edge of the aquatic vegetation mat at the time of collection (the length of the sides installed on the frame varied depending on the size of the vegetation mat). We estimated the total vegetation area as one meter (width of the area) times the average distance from where the outer edge of the vegetation met the sides of the PVC frame to the interior two stakes defining the shore. Within the area of the frame between the outer edge of the vegetation and the shore, we visually estimated the percent cover of alligatorweed, water hyacinth, water lettuce, native plant species (recorded by species when identifiable), and open water. We then calculated the estimated area of each cover category using the total area of the aquatic vegetation.

In order to estimate water depth, during each data collection we recorded the depth of the water (which could be zero) for each plot at each of the four stakes and at the edge of the aquatic vegetation. We used the water depth gauge maintained by the University of Houston, Clear Lake (∼200 m from the nearest plot) to provide another estimate of water depth (Texas Commission on Environmental Quality: gauge C734).

In October 2008, we conducted an additional field survey to examine whether the distributions of water hyacinth and alligatorweed were correlated in Armand Bayou. We haphazardly selected 104 points along the shores in the same general area as the study plots. At each point, we estimated average extent of vegetation mat and percent cover by species within an area along five meters of shoreline.

### Analysis of Data

We used repeated measures ANCOVA to examine the effects of our treatments on the abundances of water hyacinth, alligatorweed, water lettuce, native plants, and open water. Abundances were absolute abundances (square meters). Data were square-root transformed to more closely fit the assumptions of ANCOVA, such as normality.

To investigate how the presence or absence of alligatorweed affected water hyacinth establishment versus growth, we first performed a repeated measures ANCOVA in which we included all plots and the initial abundance of water hyacinth as a covariate. We then divided the overall process into several component analyses. In order to examine the effect of alligatorweed presence on water hyacinth establishment, we used an adjusted means partial difference test to examine whether the treatment combination in which both species were removed was significantly different from that in which only water hyacinth was removed (i.e. hyacinth establishing with neither hyacinth nor alligatorweed present versus its establishment with only alligatorweed present). To investigate how the presence or absence of alligatorweed affected the growth of water hyacinth patches, we performed a second adjusted means contrast test using plots in which neither species had been removed and those in which only alligatorweed had been removed (i.e. hyacinth patch growth with or without alligatorweed also present). We calculated effect sizes using Cohen’s d, to determine the net effect (facilitative or antagonistic) of the focal species removal on both establishment and average abundance of hyacinth.

To investigate the effect of water hyacinth and water lettuce on alligatorweed establishment and growth, we first performed a repeated measures ANCOVA followed by a pair of adjusted means partial difference tests focused on alligatorweed establishment (hyacinth, water lettuce, and alligatorweed removed versus only alligatorweed removed) and growth (no species removed versus hyacinth and water lettuce removed), and determination of effect sizes. Our treatments had no significant effects on the abundance of any other species (or area of open water) either as a main effect or in interaction with time so no contrast tests were performed for these other analyses.

For the 2008 natural abundance survey, we used a chi-square test to examine whether the presence of water hyacinth and alligatorweed were independent. We then used a correlation z-test to determine whether the abundances (log transformed) of these two species were correlated. Finally, we used RMA regression (model II) to determine the quantitative relationship between the abundance of these species. RMA regression differs from OLS regression in that there is not the conventional assumption of a predictor and response variable, therefore, it assumes error in both the x and y variables, and so it minimizes the Euclidian distance to the fitted line rather than the vertical distance [38].

## Results

### Water Hyacinth

**Figure 1:**
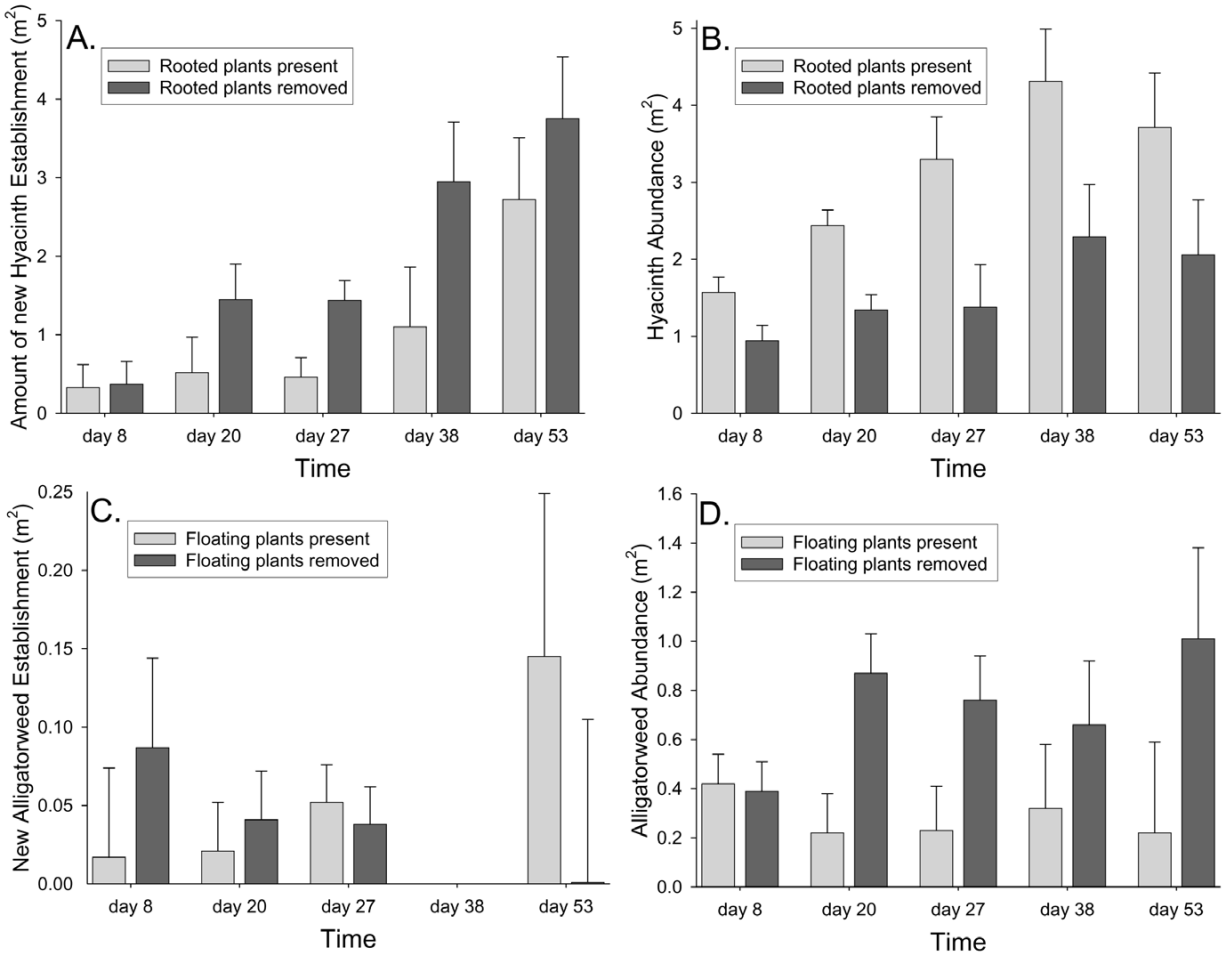
Effects of removal treatments on establishment and abundance. The effect of alligatorweed (rooted plant) presence or absence on A) establishment of water hyacinth patches (i.e. the amount of new hyacinth removed in those plots) and B) growth of water hyacinth patches. The effect of water hyacinth and water lettuce (floating plants) presence or absence on C) establishment of alligatorweed patches and D) growth of alligatorweed patches. Time is the number of days after the treatments were first imposed. Adjusted per plot means from ANCOVA with initial abundance of response species as a covariate (+1 SE).

**Figure 2:**
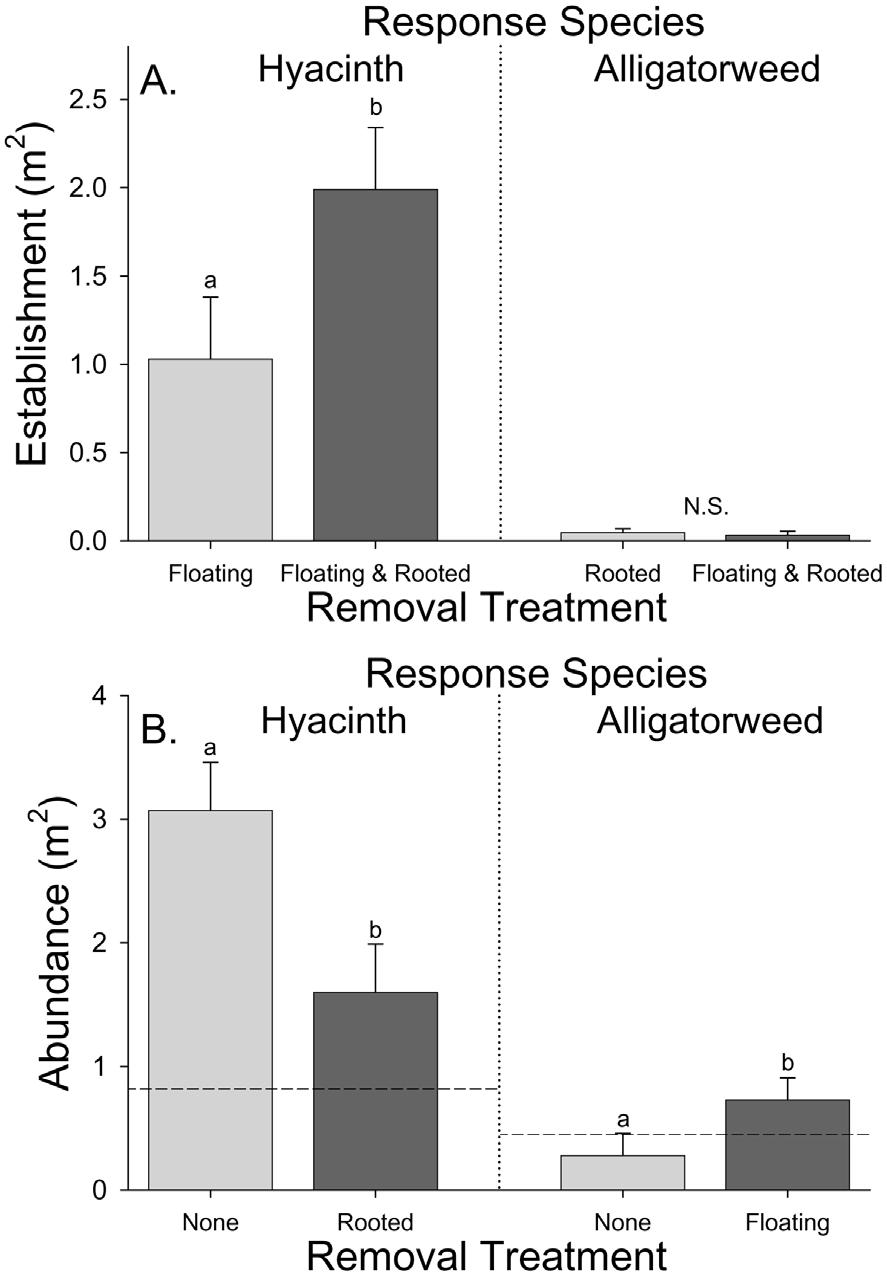
Dependence of establishment and patch growth on the presence of another species. A) Establishment of hyacinth (floating) and alligatorweed (rooted) in bare shoreline plots (floating and rooted removed) versus plots in which the other functional group was not removed (but the response species was removed). B) Abundance of hyacinth and alligatorweed in plots in which no plants were removed versus plots in which the response species was growing in plots in which the other functional group was removed. Adjusted per plot means (+1 SE) from ANCOVA with starting abundance of response species as a covariate. Letters indicate means that were significantly different in adjusted means contrast tests (four independent sets of contrast tests). Initial abundances of water hyacinth and alligatorweed are shown as dashed lines in B.

The abundance of water hyacinth depended significantly on our removal treatments (Table 1). Establishment of water hyacinth was lower in plots with alligatorweed (Table 1, Fig. 1A, 2A) but the growth of water hyacinth patches was greater in plots where alligatorweed was present than in plots where alligatorweed had been removed (Table 1, Fig. 1B, 2B). The magnitude of the negative effect of alligatorweed presence on hyacinth establishment (Cohen’s d = −2.70) was not as large as its positive effect on hyacinth patch growth (Cohen’s d = +3.76). Hyacinth abundance increased during the course of our experiment whether or not alligatorweed was present but the rate of increase was more rapid with alligatorweed present (Fig. 2B). Plots with greater initial water hyacinth abundances had higher subsequent abundances of water hyacinth and the strength of this correlation varied with sampling period. Water hyacinth abundances were independent of sampling period and the effects of removal treatments did not vary among sampling periods (Table 1).

**Table 1:** Effects of treatments on plant cover.

		Water Hyacinth		Alligatorweed		Water Lettuce		Natives		Open Water	
Factor	df	F-value	P-value	F-value	P-value	F-value	P-value	F-value	P-value	F-value	P-value
**Initial abundance**	1	**19.93**	**0.0001**	4.22	0.0645	1.16	0.6972	**39.17**	**0.0001**	2.08	0.1768
**Treatment**	3	**16.39**	**0.0310**	**5.45**	**0.0153**	0.92	0.4614	0.86	0.4909	2.25	0.1396
**Error**	11										
**Time**	4	0.71	0.5887	0.30	0.8762	1.75	0.1559	1.30	0.2865	0.84	0.5056
Time*initial abundance	4	**7.17**	**0.0002**	0.86	0.4959	0.44	0.7768	0.74	0.5685	1.28	0.2916
Time*treatment	12	1.18	0.3261	1.23	0.2929	0.49	0.9067	**2.07**	**0.0401**	1.01	0.4522
**Error (repeated)**	44										

The dependence of area of different categories of cover on aquatic plant removal treatment in repeated measures ANCOVAs with initial abundance of the response group as a covariate. Univariate tests of hypotheses for within subject effects (repeated factors) are shown in the last four rows. Significant results are shown in bold.

### Alligatorweed

Alligatorweed abundances also depended significantly on our removal treatments (Table 1). Establishment of alligatorweed did not depend on water hyacinth and water lettuce presence (Table 1, Fig. 1C, 2A) but the growth of alligatorweed patches was significantly lower when water hyacinth was present (Table 1, Fig. 1D, 2B). During the course of the experiment, the abundance of alligatorweed decreased in plots with hyacinth and water lettuce present but increased in plots with hyacinth and lettuce removed (Fig. 2B). The weak relationship between hyacinth and lettuce presence and alligatorweed establishment (Cohen’s d = +0.64) was not nearly as large as the negative effect of hyacinth and lettuce on patch growth (Cohen’s d = −2.53). Alligatorweed abundances did not depend on initial abundance, sampling period, or the interaction of sampling period with the other predictors (Table 1).

### Other Groups

Water lettuce abundances and the area of open water within the vegetation mat were independent of all predictors (Table 1). The abundance of native species depended significantly on initial abundance. This is not surprising given that many of these were rooted perennials such as *Sagittaria* spp. (duck potato), *Typha* spp. (cattails), and *Polygonum* spp. (knotweed). The abundances of native species varied significantly with the interaction of treatment and time. Visual inspection of the data suggested that high abundances of natives in hyacinth removal and control plots compared to plots with alligatorweed removal or both types of removal in the last sampling period was driving this result. Natives were independent of sampling period, treatments, and the interaction of initial abundance and sampling period.

### Water Depth

Because sampling period was not a significant predictor in any analysis, we did not analyze the water depth data further.

### Natural Abundance Survey

**Figure 3:**
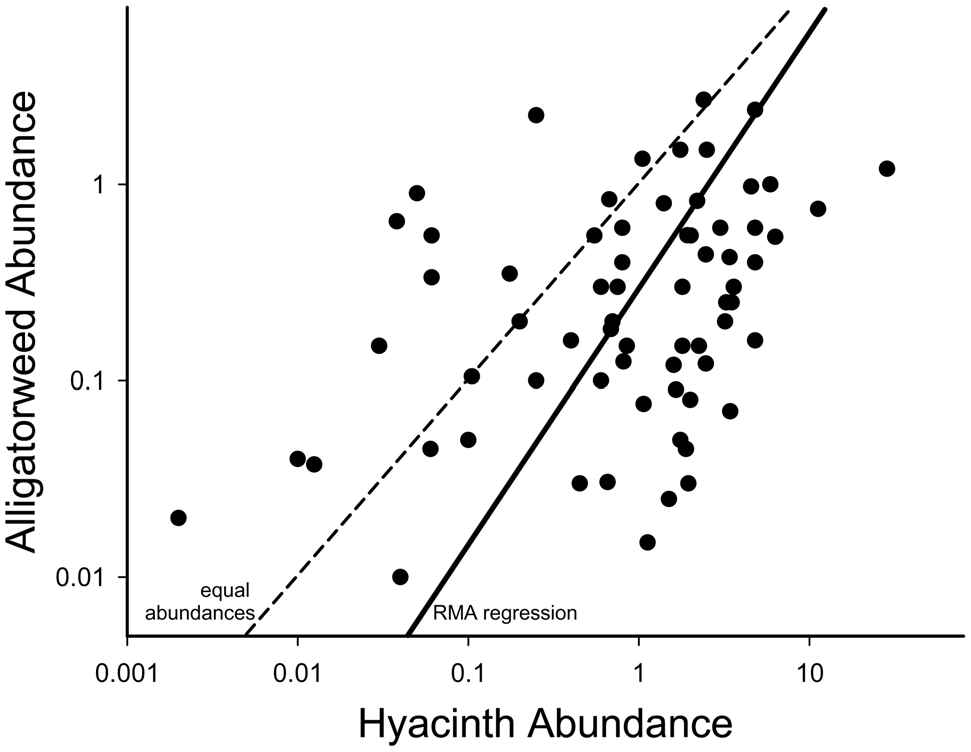
Natural abundances of alliatorweed and water hyacinth were positively correlated. Correlations between water hyacinth and alligatorweed abundances in the field survey (square meters per linear meter of shoreline). The dashed line is the unity line (equal abundances) and the solid line is the RMA regression line (y = 0.77+1.32 ×, p = 0.0009).

The presences of water hyacinth and alligatorweed were independent (chi-square = 0.42, 1 df, p = 0.52). Their abundances were significantly, positively correlated in plots in which both species occurred (r = +0.389, p = 0.0009, 69 of 104 plots). In the RMA regression, the relationship between the abundances of the two species was (Log[alligatorweed] = 0.77+1.32 Log[hyacinth], p = 0.0009). Over the range of abundances in this study, hyacinth was always more abundant on average than alligatorweed (Fig. 3).

## Discussion

The objective of this experiment was to test whether the presence or absence of exotic plants in one functional group causes successful invasions of exotic plant species in another functional group to be more likely or extensive. Our results demonstrate that distinct positive and negative interactions are occurring between exotic aquatic plant functional groups in terms of both growth and establishment. These interactions may be important in determining the species composition and extent of invasion of the exotic aquatic plant community [22], [3], [5]. Because some positive and negative effects were strong, overall invasion pressure may depend on these interactions [5]. However, we did not find mutual, strong facilitation consistent with invasional meltdown [20]. One pattern we found, a negative effect on species establishment followed by a positive effect on population growth, does not match the typical modes of succession [39] or the pattern expected during the phases of successful invasion wherein proliferation and spread are positively correlated with establishment [40], [41].

Alligatorweed decreased the establishment of water hyacinth in new areas (Fig. 2A). This may be because runners of alligatorweed that extend from shore out into open water make it more difficult for floating plants to disperse into an area where they can establish. For instance, floating plants kept farther from shore may be more subject to currents and winds. However, we observed that once water hyacinth has become established in a patch of alligatorweed, the presence of alligatorweed greatly increases the growth of water hyacinth compared to that of hyacinth growing without this underlying matrix of runners (Fig. 2B). Though alligatorweed impedes the initial establishment of water hyacinth, the overall net positive effect on water hyacinth populations is similar to a nurse plant. Tecco et al. [32] document a compelling terrestrial example of invasion in the central Argentina mountains where the exotic tree *Ligustrum lucidum* was four times as abundant under the exotic shrub *Pyracantha angustifolia* than under a native shrub, and 67 times as abundant under the exotic shrub than areas without shrub cover. However, *P. angustifolia* had varied effects on *L. lucidum*: tolerance or null effects on seedling emergence, competitive or negative effects on seedling growth, and facilitative or positive effects on sapling survival [32]. The overall effect was positive, meaning that *P. angustifolia* behaves as a nurse plant to *L. lucidum*. The fact that *L. lucidum* was also the most abundant species recruiting in the area paired with its future capacity to shade out *Pyracantha* suggests the potential for a rapid shift in exotic species abundances capable of completely changing local community composition. The similarities between this terrestrial example and our findings with aquatic exotic plants suggest that the dynamics of this aquatic plant community also have the potential to produce a rapid switch in the dynamics of the water hyacinth invasion and change local community composition.

In contrast, the presence of water hyacinth had a strong negative effect on the success of alligatorweed by hindering its growth in established areas, though it appears to have little to no effect on new establishment by alligatorweed (Fig. 2). The overall negative impact of water hyacinth on alligatorweed may shift the exotic aquatic plant community towards water hyacinth. This is consistent with the positive relationship between these two species but a greater abundance of hyacinth (Fig. 3). Water hyacinth grows in dense stands in which plants can be up to 50 cm tall [34], giving it the potential to intercept light before it reaches alligatorweed leaves on runners near the water surface [42]. However, alligatorweed also grows on the shore where it is not subject to competition with hyacinth. The inclusion of terrestrial habitats in the niche of alligatorweed and the lack of effect of water hyacinth on alligatorweed establishment may allow this invasive plant species to persist in high abundance in this ecosystem despite the apparent competitive superiority of water hyacinth in open water habitats. Alligatorweed may also persist in abundance due to its storing 10–20% of its total biomass in its roots as overwintering propagules in clonal populations, which may allow it to acquire resources and grow more quickly in the spring than some of its competitors [36].

We do not know how general these results may be in terms of interactions between different groups of exotic plants. If the crucial components are a shore-rooted plant with runners close to the water’s surface and a tall floating plant, then our findings may apply to other invasions [43]. Indeed, the lack of a significant response of water lettuce, which is far shorter than hyacinth, to alligatorweed presence suggests that the details of each species biology are likely important. In cases in which tall emergent plants interact with floating plants, the combination of an initially negative interaction becoming a positive interaction may not apply because hyacinth would not be able to shade out taller emergent macrophytes (though the effects of competition for other resources merit further consideration and study). However, our ability to tell functional group effects from idiosyncratic species effects is limited because we do not have replication within functional groups.

Though our results are at a very local scale, the interactions between water hyacinth and alligatorweed are similar to the phenomenon of alternative stable states and rapidly switching dynamics of rooted versus floating aquatic plants [44]–[46]. Since the proposed mechanism underlying high local population growth of water hyacinth in this case may include a reduction in emigration rate, the increase in hyacinth in local patches with alligatorweed may be offset by lower invasion intensity elsewhere. In fact, the high levels of hyacinth establishment in alligatorweed and hyacinth removal plots (Fig. 1A) indicate that there is a tremendous movement of hyacinth between patches. In general, a metapopulation approach to understanding floating plant invasions may lead to a greater ability to predict invasion levels at local scales and perhaps at larger spatial scales as well [47], [48]. Additionally, quantifying the effects of species interactions across multiple life stages appears necessary to determine the overall direction (positive, negative, or null) of the interaction and make the most accurate predictions [49].

This study provides useful insights for the control of exotic plants in Armand Bayou and other habitats where these species occur. First, the success of a control program may depend on the spatial extent of the control effort and may benefit from a metapopulation approach given the large amount of movement of hyacinth among patches [47], [48]. Second, the structure of shore-rooted vegetation may have a large impact on the dynamics of hyacinth population growth. Specifically, controlling alligatorweed may decrease hyacinth abundance by eliminating the positive effects of alligatorweed presence on water hyacinth population growth. Third, controlling water hyacinth may release alligatorweed from competition resulting in increased invasion of that species when hyacinth is controlled - a key consideration given the current frequency of efforts to control water hyacinth [50].

In this study, we found that alligatorweed limits the establishment of water hyacinth but increases its population growth once established and that water hyacinth limits the growth of alligatorweed. This implies simple facilitation and competition, respectively, between the invasive plant species that is not suggestive of an invasional meltdown [27]. Though research into facilitation among exotic species has accelerated greatly in the last decade, there is still much work to be done about the general mechanisms of invasions and how best to control exotic species. Identifying exotic facilitation of this nature is crucial to maximizing the efficiency and efficacy of our exotic control methods [32]. Experiments in exotic facilitation that are spatially explicit, incorporate metapopulation dynamics, or seek to discern functional group patterns despite idiosyncratic species relationships are particularly merited.
